# Hyperhomocysteinemia in Takayasu arteritis—genetically defined or burden of the proinflammatory state?

**DOI:** 10.3389/fimmu.2025.1574479

**Published:** 2025-04-04

**Authors:** Eduarda Bonelli Zarur, Faustino Peron Filho, Allan Chiaratti de Oliveira, Gerson Dierley Keppeke, Vânia D’Almeida, Alexandre Wagner Silva de Souza

**Affiliations:** ^1^ Rheumatology Division, Department of Medicine, Escola Paulista de Medicina, Universidade Federal de São Paulo, São Paulo, SP, Brazil; ^2^ Rheumatology Division, Universidade do Estado do Rio de Janeiro, Rio de Janeiro, Brazil; ^3^ Department of Pediatrics, Escola Paulista de Medicina, Universidade Federal de São Paulo, São Paulo, Brazil; ^4^ Departamento de Ciencias Biomédicas, Facultad de Medicina, Universidad Católica del Norte, Coquimbo, Chile; ^5^ Department of Psychobiology, Escola Paulista de Medicina, Universidade Federal de São Paulo, São Paulo, Brazil

**Keywords:** Takayasu Arteritis, hyperhomocysteinemia, cardiovascular disease, arterial inflammation, single nucleotide polymorphism, homocysteine, ischemic arterial events

## Abstract

Takayasu arteritis (TAK) is associated with high plasma homocysteine (Hcy) and elevated Hcy predicts ischemic events. Thus, this study aims to compare the frequency of single-nucleotide polymorphisms (SNPs) of genes involved in Hcy metabolism between TAK and controls and analyze associations with Hcy levels, TAK features, and acute ischemic arterial events (AIAEs). A cross-sectional study was performed with 73 TAK patients and 71 controls. SNPs of genes involved in the Hcy metabolism, plasma Hcy, and risk factors were analyzed for hyperhomocysteinemia (HHcy), cardiovascular disease (CVD), and AIAEs. Patients presented a higher frequency of risk factors for CVD and HHcy. At least one AIAE was observed in 27 (37.0%) patients and one control. The frequency of the SNPs was similar between both groups, and there was no association between SNP carriage and AIAEs. TAK patients presented higher Hcy levels than controls (13.9 ± 5.6 µmol/L vs. 8.6 ± 4.0 µmol/L; *p* < 0.001), and patients carrying MTHFR677TT presented higher Hcy levels than those carrying MTHFR677CT (20.4 ± 7.8 µmol/L vs. 13.7 ± 5.2 µmol/L; *p* = 0.02) or MTHFR677CC (20.4 ± 7.8 µmol/L vs. 13.1 ± 4.7 µmol/L; *p* = 0.009). TAK was an independent risk factor for HHcy [odds ratio (OR) = 10.20; 95% confidence interval (95% CI): 4.16–25.00; *p* < 0.001], and in TAK, thiazide diuretic use was a risk factor for HHcy (OR = 11.61; 95% CI: 1.63–82.63; *p* < 0.01). In conclusion, TAK was a risk factor for HHcy but not related to SNPs in genes encoding Hcy metabolism enzymes. The burden of chronic inflammation and thiazide diuretics contribute to HHcy in TAK.

## Highlights

- Higher Hcy levels in TAK are not associated with SNPs in genes of Hcy metabolism.
- TAK and thiazide diuretics *are* independent risk factors for HHcy.
- MTHFR677TT carriage is associated with higher Hcy levels in TAK.
- Hcy and SNPs in Hcy metabolism are not associated with ischemic events in TAK.

## Introduction

Takayasu arteritis (TAK) is a systemic large-vessel vasculitis affecting the aorta, its primary branches, and pulmonary arteries. TAK is a chronic disease affecting young individuals with a peak incidence around the third and fourth decades (1–3), and it is associated with high morbidity, which is especially due to cerebrovascular disease, cardiac ischemic events, and heart failure (4–6). Moreover, cardiomyopathy and ischemic stroke are the most frequent causes of death in TAK patients (7, 8) and a French study with 318 patients demonstrated that one-half of the patients will experience at least a vascular complication within 10 years after the diagnosis (9).

Homocysteine (Hcy) is an intermediate amino acid formed during the metabolism of the essential amino acid methionine. Genetic variants in enzymes involved in Hcy metabolism, nutritional deficiencies, medications such as proton pump inhibitor (PPI) and methotrexate (MTX), and comorbidities are some of the many causes of increased Hcy levels (10). Hyperhomocysteinemia (HHcy) is defined as Hcy levels higher than 10–15 μmol/L (11–13), but levels above 10 μmol/L are already associated with adverse outcomes (14, 15). The association between HHcy and cardiovascular events (CVEs) has long been demonstrated. However, its causal relation is yet to be confirmed (12, 15–18). A previous study from our group was the first to describe higher plasma Hcy levels in TAK patients compared to controls and its association with ischemic events in TAK (3). Afterward, Chen et al. confirmed the finding of higher Hcy levels in TAK patients, whereas, in contrast to our previous study, patients presenting active disease had higher Hcy levels than those in remission. In addition, higher Hcy levels were an independent risk factor for coronary artery stenosis ≥50% in TAK (19).

Although both studies have demonstrated higher Hcy levels in TAK, to date, no study has investigated factors leading to HHcy in this form of large-vessel vasculitis. Therefore, we aimed to compare the frequency of single-nucleotide polymorphisms (SNPs) of genes encoding proteins involved in the Hcy [i.e., methylenetetrahydrofolate reductase (MTHFR), methyltransferase (MTR), and methyltransferase reductase (MTRR)] and folate [i.e., reduced folate carrier (RCF-1)] metabolism pathways between patients with TAK and controls, as well as to analyze associations between Hcy levels with these SNPs, TAK features, and acute ischemic arterial events (AIAEs).

## Materials and methods

### Study design and participants

A cross-sectional study with a control group was performed. TAK patients under regular follow-up at the Universidade Federal de São Paulo Vasculitis Unit were recruited for the study. The enrollment started in July 2019, and the last patient was included in July 2023. TAK patients had to fulfill either the American College of Rheumatology 1990 criteria (20) or Ishikawa diagnostic criteria modified by Sharma (21). All participants had to be at least 18 years old and were excluded if they presented end-stage renal disease. Participants from the control group were recruited from non-relative companions of patients followed by the Vasculitis Outpatient Clinic. Controls were excluded if they had any systemic inflammatory or autoimmune disease.

### Study assessments

The following data were recorded from both groups: demographic and anthropometric data; a previous history of AIAE; risk factors for cardiovascular disease (CVD) such as arterial hypertension (HTN), smoking status, diabetes mellitus, and sedentary lifestyle; known vitamin deficiencies or diseases related to vitamin deficiencies including atrophic gastritis, hypothyroidism, and alcoholism; history of chronic kidney disease and concomitant use of medications that may interfere with Hcy levels, e.g., MTX, PPI, nicotinic acid, and fibrates. The AIAEs analyzed were stroke and transient ischemic attack (TIA), myocardial infarction, angina pectoris, mesenteric angina, and peripheral ischemia according to established definitions (22–26). Study participants with less than 3 hours of aerobic exercise weekly for at least 2 months were considered to have a sedentary lifestyle (27). In the TAK group, we analyzed age at diagnosis, disease duration, and current therapy, while disease activity was evaluated by Kerr’s criteria (28). Finally, laboratory data performed routinely by the patients (e.g., lipid and glycemic profiles, erythrocyte sedimentation rate, creatinine, B12 vitamin, and folic acid) were retrieved from medical records.

### Laboratory tests

Briefly, blood sampling was performed by collecting 6 mL of peripheral blood in ethylenediaminetetraacetic acid (EDTA) tubes for DNA extraction and Hcy measurement. As previously reported, plasma Hcy was measured by high-performance liquid chromatography (29). The DNA was extracted using the *FlexiGene^®^ DNA* (ref 51206, Qiagen^®^, Hilden, Germany) extraction kit, following the manufacturer’s instructions. Gene fragments of interest were amplified by polymerase chain reaction (PCR) using the primers presented in **Supplementary Table S1**, with the *Phusion Flash High-fidelity PCR Master Mix^®^* (ref F-548 Thermo Fisher^®^, Waltham, MA, USA). Fragment sizes were confirmed by agarose gel. All reference sequences were extracted from the National Center for Biotechnology Information (NCBI) gene database (**Supplementary Table S1**). Finally, SNP sequencing was performed using the Sanger technique (30) with either the forward or reverse primer. The following SNPs and their respective genes of proteins involved in the Hcy and folate metabolism were evaluated in the study: C677T (rs1801133) and A1298C (rs1801131) in the *MTHFR* gene, A2756G (rs1805087) in the *MTR* gene, A66G (rs1801394) in the *MTRR* gene, and G80A (rs1051266) of the *SLC19A1* gene.

### Statistical analysis

The sample size was estimated as 70 individuals in each group, and it was based on a possible difference of 25% in the MTHFRC677T SNP between TAK patients and controls. The alpha error was 5% with a power of 90%.

Categorical variables are presented by percentage and absolute number, while continuous variables are presented by mean ± (SD) or median [interquartile range (IQR)], according to the distribution. Categorical data were compared between groups by Fisher’s exact test or chi-square test. Continuous data were compared by Student’s t-test or Mann–Whitney U test, and the Kruskal–Wallis test or one-way ANOVA, according to the number of groups and variable distribution. The Mann–Whitney U test and Scheffe’s test were performed for the *post-hoc* analysis of the Kruskal–Wallis and one-way ANOVA, respectively. Hcy was evaluated as a continuous variable as well as a categorical variable, with a cut-off of 10 μmol/L, according to previous studies (14, 17, 18). Two models of multivariate binary logistic regression were built to assess predictors of HHcy in all participants including the SNPs of genes involved in the Hcy and folate metabolism, TAK, and some risk factors for CVD and HHcy as independent variables. A third logistic regression model included only TAK patients, and its independent variables were HTN, anti-hypertensive drugs, and other drugs related to HHcy. Results are presented as an odds ratio (OR) with a 95% confidence interval (95% CI).

The Hardy–Weinberg equilibrium in analyzed genes was tested by the chi-square test with one degree of freedom with the R software version 4.3.2. Statistical analysis was performed using the IBM SPSS software for Windows v. 21.0 (Armonk, NY, USA), and graphs were built using the GraphPad Prism for Windows v. 9.0 (San Diego, CA, USA). A *p*-value of <0.05 was considered significant. For multiple *post-hoc* comparisons, the adjusted *p*-value was 0.01 according to Bonferroni’s correction.

## Results

### Patients with TAK and controls

We included 144 participants in the study: 73 in the TAK group and 71 in the control group. Most of the participants were female in both groups (95.9% vs. 94.3%; *p* = 0.67), and the median age was also similar between TAK and controls [43.0 years (32.0–50.5) vs. 41.0 years (33.5–53.5); *p* = 0.53]. However, the groups differed in self-reported race, with a higher frequency of White persons in controls and Mestizos predominant among TAK patients (*p* < 0.001 and *p =* 0.003, respectively). The median time since TAK diagnosis was 120.0 months (48.0–204.0).

Patients with TAK showed a higher frequency of risk factors for CVD such as obesity (36.8% vs. 19.1%; *p* = 0.02), sedentary lifestyle (78.6% vs. 50.7%; *p* = 0.001), and HTN (82.2% vs. 11.6%; *p* < 0.001) than controls, whereas the frequencies of diabetes (12.3% vs. 10.1%; *p* = 0.68) and current smoking (5.5% vs. 1.4%; *p* = 0.38) were similar between both groups. Regarding the risk factors for HHcy, only TAK patients used MTX (24.7%), and more TAK patients used PPI than controls (38.4% vs. 4.3%; *p* < 0.001). However, no differences were found in the frequency of hypothyroidism (6.8% vs. 14.5%; *p* = 0.14) or metformin use (15.1% vs. 10.1%; *p* = 0.14).

Among protective factors for HHcy, only TAK patients were under acetylsalicylic acid (ASA) therapy (83.6%), while statin was more frequently used by TAK patients than controls (63.0% vs. 10.2%; *p* = 0.001). Folic acid was taken by 28.8% of TAK patients and 5.8% of the controls (*p* < 0.001). However, most of the TAK patients taking folic acid were also under MTX therapy. The use of B-complex vitamins was similar between the TAK patients and controls (2.7% vs. 4.3%; *p* = 0.60).

Twenty-seven (37.0%) patients experienced at least one AIAE, and most of them had angina pectoris (48.1%), stroke (25.9%), or myocardial infarction (22.2%). Abdominal angina was observed in only 14.8% of the patients, and one patient had a previous TIA. No cases of peripheral ischemia were observed. Only one AIAE (i.e., stroke) was found in the control group.

### Homocysteine levels and TAK characteristics

**Figure 1:**
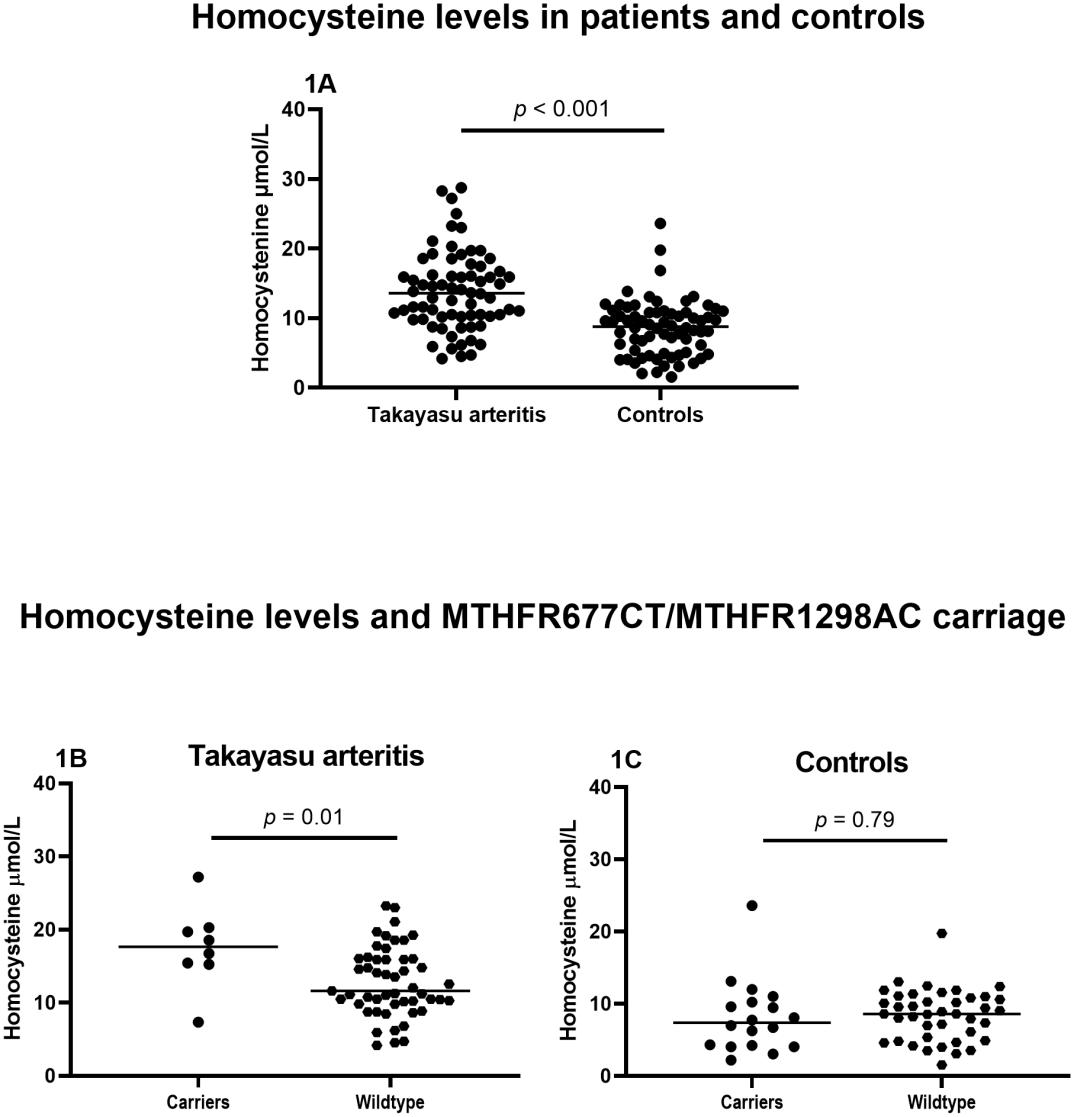
Plasma homocysteine levels among TAK patients and controls and according to different *MTHFR* genotypes. Legend: TAK, Takayasu arteritis. The transverse bar represents the mean concentration of homocysteine. **(A)** Homocysteine levels among patients and controls. **(B)** Homocysteine levels among patients carrying MTHFR677CT and MTHFR1298AC vs. wild type. **(C)** Homocysteine levels among controls carrying MTHFR677CT and MTHFR1298AC vs. wild types.

Mean Hcy levels were significantly higher in TAK patients than in controls (13.9 ± 5.6 µmol/L vs. 8.6 ± 4.0 µmol/L; *p* < 0.001) (**Figure 1A**). Hyperhomocysteinemia (i.e., plasma Hcy >10 µmol/L) was observed in 82.5% of hypertensive patients with TAK, while only 53.8% of those without HTN presented HHcy (*p* = 0.03). Such a difference was not found in the control group. Furthermore, HHcy was more frequently presented in patients using hydrochlorothiazide than those without it (42.6% vs. 12.5%; *p* = 0.003), while no differences in Hcy levels were found regarding the use of other anti-hypertensive drugs such as angiotensin-converting enzyme inhibitors, angiotensin receptor blockers, calcium channel blockers, hydralazine, and spironolactone, as well as statins or furosemide (**Supplementary Table S3**).

Patients with previous AIAEs had lower Hcy levels than those without [12.0 ± 4.6 µmol/L vs. 14.8 ± 5.9 µmol/L; *p* = 0.04), whereas there was no difference in the frequency of AIAEs between patients with and without HHcy (29.6% vs. 50.0%; *p* = 0.13). Finally, patients in remission tended to present lower Hcy levels than those with active disease [11.5 ± 5.3 µmol/L vs. 14.6 ± 5.6 µmol/L; *p* = 0.05].

### Genetic polymorphism frequencies and Hcy levels

In both groups, all genes encoding enzymes involved in Hcy metabolism were in Hardy–Weinberg equilibrium, while only in TAK patients was the *SLC19A1* gene encoding the protein reduced folate carrier-1 (RFC-1) not in Hardy–Weinberg equilibrium (x^2^ = 6.829; *p* = 0.03). The frequencies of the SNPs of genes encoding enzymes involved in the Hcy (i.e., MTHFR, MTR, and MTRR) and folate (i.e., RCF-1) metabolic pathways were similar between TAK patients and controls (**Table 1**) and between TAK patients with and without previous AIAEs (**Supplementary Table S4**). Plasma Hcy levels were higher in patients carrying the MTHFR677TT genotype compared to the CT genotype (*p* = 0.02) and CC (*p* = 0.01) and among those carrying both *MTHFR* SNPs (i.e., *MTHFR* C677T and *MTHFR* A1298C) in heterozygosis compared to both *MTHFR* wild types (**Figures 1B, C**). Conversely, no difference was found in Hcy levels between all individual SNPs in controls (**Table 2**).

**Table 1:** The frequency of specific genotypes between TAK patients and controls.

Variables	TAK (n = 73)	Controls (n = 71)	*p*
MTHFR677TT	6 (8.3)	7 (10.1)	0.52
MTHFR677CT	32 (44.4)	36 (52.2)	
MTHFR677CC	34 (47.2)	26 (37.7)	
MTHFR1298CC	6 (8.5)	2 (4.0)	0.29
MTHFR1298AC	27 (38.0)	32 (45.7)	
MTHFR1298AA	38 (53.5)	36 (51.4)	
MTR2756GG	2 (2.8)	6 (8.8)	0.27
MTR2756AG	24 (33.3)	24 (35.3)	
MTR2756AA	46 (63.9)	38 (55.9)	
MTRR66GG	13 (20.3)	15 (21.1)	0.88
MTRR66AG	36 (56.3)	37 (52.1)	
MTRR66AA	19 (26.8)	15 (23.4)	
SLC19A1 80AA	9 (13.0)	13 (21.0)	0.39
SLC19A1 80AG	45 (65.2)	34 (54.8)	
SLC19A1 80GG	15 (21.7)	15 (24.2)	

There are a few missing values in different SNPs. Data presented as n (%).

TAK, Takayasu arteritis; MTHFR, methylenetetrahydrofolate reductase; MTR, methyltransferase; MTRR, methyltransferase reductase; SNPs, single-nucleotide polymorphisms; SLC19A1, solute carrier family 19 member 1; n, total number of participants in each group.

**Table 2:** Homocysteine serum levels among different genotypes.

Hcy, μmol/L	Wild type	Heterozygous	Homozygous for the MAF	*p*
TAK				
MTHFR677	13.0 ± 4.7	13.7 ± 5.2	20.4 ± 7.8	0.01*
MTHFR1298	11.6 (9.8–15.9)	15.4 (10.8–18.6)	14.9 (9.3–22.3)	0.32
MTR2756	14.1 ± 5.7	13.8 ± 5.5	NA	NA
MTRR66	14.8 ± 5.1	13.4 ± 5.8	14.8 ± 5.4	0.58
SLC19A1	15.8 ± 5.7	13.5 ± 5.7	14.8 ± 4.0	0.37
Controls				
MTHFR677	8.5 ± 3.6	8.4 ± 4.3	8.7 ± 3.5	0.99
MTHFR1298	8.5 ± 3.5	8.7 ± 4.6	NA	NA
MTR2756	8.1 ± 4.0	8.8 ± 4.5	9.8 ± 3.1	0.61
MTRR66	7.5 ± 3.6	7.9 ± 2.9	10.0 ± 5.5	0.17
SLC19A1	8.8 ± 5.7	8.3 ± 3.8	8.0 ± 2.8	0.88

Results are presented as mean and standard deviation or as median and interquartile range.

Hcy, homocysteine; TAK, Takayasu arteritis; NA, not applicable; MAF, minor allele frequency; MTHFR, methylenetetrahydrofolate reductase; MTR, methyltransferase; MTRR, methyltransferase reductase; SLC19A1, solute carrier family 19 member 1.

*Flags significant results.

### Analyses of predictors of HHcy

We built multivariate binary logistic regression analysis to assess predictors for HHcy, defined as Hcy levels higher than 10 μmol/L. The first model included the SNPs of genes involved in the Hcy and folate metabolism pathways and TAK. TAK increased 10 times the risk of presenting HHcy, regardless of the carriage of any of the individual SNPs. We performed another logistic regression model, including the genotype MTHFR677TT, some CVD and HHcy risk factors, and TAK. TAK persisted as an independent risk factor for HHcy in this model (**Table 3**). Since TAK was found to be an independent risk factor for HHcy, we performed a third multivariate binary logistic regression model including only TAK patients to analyze predictors for HHcy. In this model, HTN, anti-hypertensive drugs, and other drugs typically used for the treatment of TAK, which are known to interfere with plasma Hcy levels, were the independent variables. We found that thiazide diuretic use was the only predictor for HHcy in TAK patients, with an 11-fold increase in the risk of HHcy (**Table 3**).

**Table 3:** Binary logistic regression models for HHcy predictors.

Variables	OR	95% CI	*p*
Model 1			
TAK	10.20	4.16–25.00	<0.001*
MTHFR677TT	1.89	0.42–8.55	0.41
MTHFR1298CC	3.81	0.34–42.20	0.28
MTR2756GG	4.19	0.63–27.92	0.14
MTRR66GG	3.09	0.99–9.63	0.05
SLC19A1AA	1.46	0.48–4.46	0.50
Model 2			
TAK	4.28	1.40–13.03	0.01*
MTHFR677TT	2.08	0.50–8.61	0.31
HTN	2.03	0.60–6.84	0.25
Diabetes	0.81	0.23–2.87	0.74
Obesity	0.71	0.27–1.86	0.49
Hypothyroidism	0.50	0.13–1.89	0.31
Model 3			
Thiazide diuretics	11.61	1.63–82.63	0.01*
HTN	2.09	0.41–10.79	0.38
ACEi	4.67	0.68–32.12	0.12
Beta-blockers	0.37	0.08–1.60	0.18
ASA	0.11	0.01–1.10	0.06
PPI	1.99	0.51–7.71	0.32
MTX	1.56	0.36–6.78	0.56

ACEi, angiotensin-converting enzyme inhibitors; ASA, acetylsalicylic acid; HTN, hypertension; MTHFR, methylenetetrahydrofolate reductase; MTR, methyltransferase; MTRR, methyltransferase reductase; MTX, methotrexate; OR, odds ratio; PPI, proton pump inhibitor; SLC19A1, solute carrier family 19 member 1; SNP, single-nucleotide polymorphism; TAK, Takayasu arteritis; HHCy, hyperhomocysteinemia (i.e., Hcy > 10 μmol/L).

*Flags significant results.

## Discussion

This is the first study to address possible mechanisms involved in the HHcy described in TAK. Indeed, we confirm that HHcy is more frequently observed in TAK compared to those without TAK, and we identified TAK *per se* as an independent risk factor for HHcy, increasing its risk by 10-fold. Furthermore, among TAK patients, thiazide diuretic use was associated with an 11-fold increase in the risk of presenting HHcy. Nevertheless, we did not find differences regarding the frequency of Hcy and folate metabolism-related SNPs between TAK patients and controls, reinforcing that TAK-associated HHcy may be due to the pro-inflammatory state observed in TAK or therapy, but not genetically inherited.

In this study, traditional cardiovascular risk factors (e.g., obesity, smoking, sedentary lifestyle, and HTN) were more frequent among TAK patients. In line with our findings, previous studies detected a higher prevalence of HTN (27, 31–33), metabolic syndrome (27), and dyslipidemia (27) in TAK. A previous study from our group also found a higher prevalence of HTN and hypertriglyceridemia in TAK patients compared to controls. Still, the frequency of obesity and smoking was not higher in TAK. Indeed, other studies could not find an increased frequency of smoking or obesity in TAK (31, 32).

TAK patients present a higher frequency of CVE than the general population, and they represent the main cause of mortality in TAK (7, 34, 35). Nevertheless, the burden of risk factors for cardiovascular disease does not thoroughly explain the higher frequency of CVE in TAK, and available risk estimation tools do not seem to estimate accurately cardiovascular risk in TAK as they estimate in the general population (31, 34, 35). Disease-related factors such as accelerated atherosclerosis, regardless of cardiovascular risk factors, arterial damage from previous inflammation, and Hata and Numano angiographic type V, seem to contribute to the CVE burden in TAK (31, 32, 34, 35).

Hyperhomocysteinemia has long been associated with cardiovascular events. However, its causal relationship is yet to be confirmed (12, 15–18). In this study, we confirm previous findings of higher Hcy levels in TAK patients (3, 19). In contrast to our results, previous studies had found an association between HHcy and acute ischemic events (3) and coronary artery involvement in TAK (19), while in the present study, patients with previous AIAEs had surprisingly lower Hcy levels than patients without AIAEs. The smaller sample size of the first study (N = 29) (3) compared to this one (N = 73) may have contributed to that different finding regarding ischemic events, and we speculate if the treatment required after a first CVE could contribute to lower Hcy levels. As for coronary involvement, we did not perform routinely coronary angiography or CTA to assess possible asymptomatic coronary involvement, and asymptomatic patients with coronary artery involvement may have been missed in our study (19). Nevertheless, Hcy levels higher than 10 μmol/L were not associated with an increased frequency of AIAEs in TAK patients, and only 16 patients presented Hcy levels equal to or lower than 10 μmol/L, which is the cut-off point already associated with higher adverse outcomes of HHcy (14, 15).

We observed that most hypertensive TAK patients presented HHcy. Similarly, previous studies have demonstrated that HHcy increases the risk of presenting HTN (17, 36) and that patients with HTN carrying the MTHFR677T allele had higher Hcy levels than those without HTN (17). Furthermore, a 5 µmol/L increase in plasmatic Hcy was associated with a 50% increase in HTN risk among women (37), and those patients who present HTN associated with Hcy levels >10 μmol/L are regarded as “H-HTN” (14, 38). However, other studies have failed to find associations between HHcy and increased risk of HTN (39, 40). Patients with H-HTN seem to have an increased risk of stroke (14, 36), and a mega trial including 20,000 Chinese hypertensive patients demonstrated that therapy with anti-hypertensive drugs associated with folic acid supplementation prevented primary neurovascular events (41).

In this study, we found a higher frequency of HHcy among patients treated with hydrochlorothiazide and that thiazide diuretic use was an independent risk factor for HHcy in TAK patients, increasing the risk by 11-fold. Indeed, it has been previously described that diuretics in general (42) and specifically hydrochlorothiazide (43, 44) use may increase Hcy plasma levels, while beta-blockers and angiotensin-converting enzyme (ACE) inhibitors have been associated with a reduction in Hcy levels (43, 44). In line with this, we observed that thiazide diuretics were the only antihypertensive agents interfering with plasma Hcy. One may argue that hydrochlorothiazide is usually the first-line agent prescribed as an antihypertensive treatment and that this could contribute as a confounding factor to the association of HHcy and thiazide diuretics, as well as between HHcy and HTN. Conversely, only 27 out of the 60 hypertensive patients analyzed in this study were treated with a thiazide diuretic. Moreover, in the multivariate analysis including HTN and thiazide diuretic use, the latter remained as the only predictor of HHcy. Thus, our study suggests that there may be an association between HHcy and thiazide diuretic use in TAK, and further studies are required to unravel if there is a causal relationship and if it is of clinical relevance.

The frequency of the genotypes was similar between TAK patients and controls, suggesting that TAK-associated HHcy is not due to genetic factors. Only in the TAK group were Hcy levels higher in patients presenting the MTHFR677 minor allele in homozygosis (i.e., MTHFR677TT) compared to other MTHFR677 genotypes, as well as in those carrying both *MTHFR* SNPs (i.e., *MTHFR* C677T and *MTHFR* C1298A) in heterozygosis compared to both wild types. One possible explanation for this finding is the folic acid fortification policy in Brazil, which possibly attenuates the HHcy observed by the carriage of these SNPs (45). We speculate that TAK-associated HHcy mechanisms may overpower the effect of folic acid fortification, leading to higher Hcy levels upon MTHFR677 minor allele carriage in homozygosis. In line with that, we demonstrated that TAK *per se* independently increased 10 times the risk for HHcy. HHcy has long been associated with inflammatory states, but the mechanism through which it occurs remains poorly elucidated. One hypothesis is that the chronic inflammatory states would lead to a reduction in pyridoxal phosphate (PLP) bioavailability (i.e., the active form of vitamin B6) due to its recruitment to inflammatory sites. PLP has a role in reactions involving immunotolerance and modulation, suppression of inflammation, and proliferation of immune cells (46). The lower availability of PLP, which is a cofactor of cystathionine B-synthase (CBS), could lead to reduced activity of CBS, culminating in higher Hcy levels (47). It has been demonstrated in observational studies that IL-6 and IL-1ra levels were strongly and independently correlated with HHcy (48) and that IL-6 and PLP levels were negatively correlated (49). In addition, an intervention study with rheumatoid arthritis demonstrated that vitamin B6 supplementation was able to suppress IL-6 and TNF-alpha production (50).

Finally, it is of utmost importance to further explore the relationship between TAK, HHcy, HTN, thiazide diuretic use, and CVE, especially cerebrovascular events, in prospective studies due to the following reasons: CVEs are the main cause of mortality among TAK patients (7, 8), HHcy may be associated with CVE (12, 15–18), H-HTN increases the risk of stroke (14, 36), HTN is highly prevalent among TAK patients (27, 31–33), TAK is an independent risk factor for HHcy, and thiazide diuretic use is an independent risk factor for HHcy in TAK patients. Hence, HHcy may be a biomarker or act synergically with HTN to increase the cardiovascular risk in TAK. Stricter HTN treatment and HHcy-lowering interventions may be of benefit in preventing cerebrovascular events. It must be further investigated whether the thiazide diuretic’s increment on Hcy levels is indeed clinically relevant to tailoring its use by plasma Hcy levels in TAK patients.

Our study has some limitations; first, its cross-sectional design impairs the inference of causality between HHcy and SNP carriage, thiazide diuretic use, or TAK status and features. Furthermore, some data were retrospectively collected through chart review and patient interviews, which may have been subject to recall bias. Moreover, the TAK criteria used to select patients were not uniform, and there was a significant difference in self-reported race between TAK patients and the control group, both of which could have influenced the results. Another limitation that should be acknowledged in this study is its relatively small sample size, which is commonly seen among TAK studies since it is a rare disease. However, the sample size estimated for the most common SNP (i.e., MTHFR677) was achieved. Finally, one of the studied genes was not in Hardy–Weinberg equilibrium among patients, which may also have influenced the results.

Our study confirms the higher Hcy levels presented by TAK patients. However, it refutes the association between HHcy and the carriage of SNPs of genes involved in Hcy and folate metabolism, and we speculate that it may possibly be due to the pro-inflammatory state of TAK patients. The TAK status increases the risk of HHcy, and among TAK patients, thiazide diuretic use and the carriage of the MTHFR677 minor allele are associated with a higher frequency of HHcy. Further studies are necessary to unravel these associations and to investigate the effect of folic acid supplementation on plasma Hcy levels in TAK patients, particularly those at higher risk for HHcy, such as those using thiazide diuretics.

## Data Availability

The raw data supporting the conclusions of this article will be made available by the authors, without undue reservation.
