# Supplementary material for: Hyperhomocysteinemia in Takayasu arteritis—genetically defined or burden of the proinflammatory state?
Source: Front Immunol. 2025 Apr 4;16:1574479. doi: 10.3389/fimmu.2025.1574479 (PMC12006095; doi:10.3389/fimmu.2025.1574479)
Supplement: Supplementary file 1 [file DataSheet1.docx]

**Supplementary material**

| **Gene** | **NCBI reference codes** | **Forward primer sequence** | **Reverse primer sequence** |
| --- | --- | --- | --- |
| MTHFR677 | NG_013351 | TGAAGGAGAAGGTGTCTGCGGGA | AGGACGGTGCGGTGAGAGTG* |
| MTHFR1298 | NG_013351 | CTTTGGGGAGCTGAAGGACTACTAC | GAGGTCTCCCAACTTACCCTTCTC* |
| MTR2756 | NG_008959 | TGTTCCCAGCTGTTAGATGAAAATC | GATCCAAAGCCTTTTACACTCCTC* |
| MTRR66 | NG_008856 | GGCTCATTTGAGATTAGTGCTG* | GTGAAGATCTGCAGAAAATCCATGTA |
| SLC19A1 | NG_028278 | AGTGTCACCTTCGTCCCCTC* | CTCCCGCGTGAAGTTCTT |

**Supplementary Table S1 - NCBI reference sequences of the SNPs assessed in this study and primers sequences used for the polymerase chain reaction.**

NCBI – National Center for Biotechnology Information; MTHFR – Methylenetetrahydrofolate Reductase; MTR – methyltransferase; MTRR - methyltransferase reductase; SLC19A1 - solute carrier family 19 member 1; SNPs – Single nucleotide polymorphisms. *Highlights primers used for SNP sequencing.

**Supplementary Table S2 - Demographic data of the study’s participants.**

| **Variables** | **TAK**  **(n = 73)** | **Controls**  **(n = 71)** | ***p*** |
| --- | --- | --- | --- |
| Age, years | 43.0  (32.0-50.5) | 41.0  (33.5 – 53.5) | 0.53 |
| Females, n (%) | 70 (95.9) | 67 (94.3) | 0.67 |
| Race |  |  | 0.002*# |
| Whites, n (%) | 40 (56.3) | 59 (85.5) | 0.001* |
| Blacks, n (%) | 9 (12.7) | 4 (6.4) | 0.161 |
| Mestizos, n (%) | 20 (28.2) | 6 (8.7) | 0.003* |
| Asians, n (%) | 2 (2.8) | 0 (0.0) | NA |

TAK – Takayasu arteritis; n – Number of study’s participants; * - Flags significant results; # - p value for the overall analysis regarding race between patients and controls.

**Supplementary Table S3 – Frequency of HHcy according to anti-hypertensive drugs and statins use.**

| **Variables** | **HCY ≥ 10 μmol/L** | **HCY < 10 μmol/L** | ***p*** |
| --- | --- | --- | --- |
| Hydrochlorothiazide, n (%) | 23 (42.6) | 2 (12.5) | 0.03***** |
| ACEi, n (%) | 13 (24.1) | 2 (12.5) | 0.49 |
| ARB, n (%) | 18 (33.3) | 4 (25.0) | 0.53 |
| CCB, n (%) | 24 (44.4) | 8 (50.0) | 0.69 |
| Hydralazine, n (%) | 5 (9.3) | 1 (6.3) | 1.00 |
| Spironolactone, n (%) | 7 (13.0) | 7 (6.3) | 0.67 |
| Furosemide, n (%) | 7 (13.0) | 1 (6.3) | 0.67 |
| Statins, n (%) | 36 (66.7) | 8 (50.0) | 0.25 |

ACEi - angiotensin-converting enzyme inhibitors; ARB - angiotensin receptor blockers; CCB – calcium channel blockers; HHCy – hyperhomocysteinemia (> 10 μmol/L); TAK – Takayasu arteritis; **p* < 0.05

**Supplementary Table S4 – Comparisons between patients with and without AIAE regarding the carriage of specific SNPs.**

| **Variables** | **Acute ischemic arterial event**  **(n = 27)** | **Without acute ischemic arterial event**  **(n = 46)** | ***p*** |
| --- | --- | --- | --- |
| MTHFR 677TT, n (%) | 0 (0.0) | 6 (13.0) | 0.08 |
| MTHFR 1298CC, n (%) | 3 (12.0) | 3 (6.5) | 0.66 |
| MTR 2756GG, n (%) | 0 (0.0) | 2 (4.4) | 0.53 |
| MTRR 66GG, n (%) | 5 (19.2) | 10 (22.2) | 0.77 |
| SLC19A1 80AA, n (%) | 3 (12.0) | 6 (13.6) | 1.00 |

AIAE – Acute ischemic arterial event; MTHFR – Methylenetetrahydrofolate Reductase; MTR – methyltransferase; MTRR - methyltransferase reductase; n – Number of patients; SLC19A1 - solute carrier family 19 member 1.
